# Supplementary material for: Analysis of PPARγ Signaling Activity in Psoriasis
Source: Int J Mol Sci. 2021 Aug 10;22(16):8603. doi: 10.3390/ijms22168603 (PMC8395241; doi:10.3390/ijms22168603)
Supplement: Supplementary file 1 [file ijms-22-08603-s001.zip › Supplemental materials_Analysis of PPARg signaling activity in psoriasis/Pathway models/Models images and html files/PPARG inhibits phenotypes assotiated with psoriasis/142857.html]

cell interaction


# Cell Process cell interaction

|  |  |
| --- | --- |
| URN | urn:agi-cellproc:cell%20interaction |
| Total Entities | 83 |
| Connectivity | 3171 |
| Name | cell interaction |

---

|  |  |
| --- | --- |
| ChildConcepts | leukocyte interaction |
|  | T-cell interaction |
|  | platelet interaction |
|  | macrophage interaction |
|  | monocyte adhesion |
|  | donor-host cell interaction |
|  | leukocyte-endothelial cell interaction |
|  | microglia interaction |
|  | B-cell interaction |
|  | immunocompetent cell interaction |
|  | stem cell interaction |
|  | endothelial cell interaction |
|  | neutrophil interaction |
|  | phagocyte interaction |
|  | dendritic cell interaction |
|  | follicular dendritic cell interaction |
|  | eosinophil interaction |
|  | natural killer cell interaction |
|  | antigen-presenting cell interaction |
|  | alveolar macrophage interaction |
|  | bone marrow stromal cell interaction |
|  | granulocyte interaction |
|  | Kuppfer cell interaction |
|  | plasmacytoid dendritic cell interaction |
|  | haematopoietic stem cell interaction |
|  | mast cell interaction |
|  | cancer stem cell interaction |
|  | CD4+ T-cell interaction |
|  | lymphocyte interaction |
|  | myofibroblast interaction |
|  | regulatory T-cell interaction |
|  | CD8+ T-cell interaction |
|  | myeloid cell interaction |
|  | helper T-cell interaction |
|  | lymphoid progenitor cell interaction |
|  | plasma cell interaction |
|  | fibroblast interaction |
|  | dermal microvascular endothelial cell interaction |
|  | myeloid derived suppressor cell interaction |
|  | umbilical vein endothelial cell interaction |
|  | activated T-cell interaction |
|  | activated monocyte interaction |
|  | megakaryocyte interaction |
|  | cytotoxic T-cell interaction |
|  | capillary endothelial cell interaction |
|  | Langerhans cell interaction |
|  | natural killer T-cell interaction |
|  | megakaryocyte progenitor cell interaction |
|  | memory B-cell interaction |
|  | erythroblast interaction |
|  | conventional dendritic cell interaction |
|  | follicular B-cell interaction |
|  | decidual natural killer cell interaction |
|  | erythroid cell interaction |
|  | bone marrow derived mesenchymal stem cell interaction |
|  | hematopoietic cell interaction |
|  | PBMC interaction |
|  | immune system cell interaction |
|  | invariant natural killer T-cell interaction |
|  | Tfh cell interaction |
|  | Th0 cell interaction |
|  | effector T-cell interaction |
|  | basophil interaction |
|  | memory T-cell interaction |
|  | activated CD4+ T-cell interaction |
|  | cancer-associated fibroblast interaction |
|  | tumor-infiltrating lymphocyte interaction |
|  | promyelocyte interaction |
|  | thymocyte interaction |
|  | germinal center B-cell interaction |
|  | mononuclear phagocyte interaction |
|  | Th1 cell interaction |
|  | naive T-cell interaction |
|  | activated dendritic cell interaction |
|  | bone marrow derived mast cell interaction |
|  | bone marrow macrophage interaction |
|  | regulatory B-cell interaction |
|  | senescent T-cell interaction |
|  | Th2 cell interaction |
|  | brain capillary endothelial cell interaction |
|  | tumor infiltrating macrophage interaction |
|  | mononuclear leukocyte interaction |
|  | inflammatory monocyte interaction |

---

|  |  |
| --- | --- |
| Pathway | PPARG inhibits phenotypes assotiated with psoriasis |
|  | Neighbors of cell interaction |

---

|  |  |
| --- | --- |
| MedScan ID | 4203601 |

---

|  |  |
| --- | --- |
| Alias | cell interaction |
|  | cell cell interactions |
|  | cell interactions |
|  | cell cell interaction |
|  | cellular interaction |
|  | cellular interactions |

---
